# *In vitro* and *in vivo* evaluation of liposomes modified with polypeptides and red cell membrane as a novel drug delivery system for myocardium targeting

**DOI:** 10.1080/10717544.2020.1754525

**Published:** 2020-04-20

**Authors:** Xueyan Liu, Liangke Zhang, Wengao Jiang, Zhangyou Yang, Zongjie Gan, Chao Yu, Ran Tao, Huali Chen

**Affiliations:** a College of Pharmacy, Chongqing Medical University, Chongqing, China;; b Chongqing Research Center for Pharmaceutical Engineering, Chongqing Medical University, Chongqing, China;; c Research Center for Innovative Pharmaceutical and Excipient Analysis Technology, Chongqing Medical University, Chongqing, China

**Keywords:** Myocardium delivery, liposomes, TAT, PCM, red cell membrane

## Abstract

Ischemic cardiac disease (ICD) is a cardiovascular disease with high morbidity and mortality. In this study, a novel myocardial targeted drug delivery system was developed represented by co-modified liposomes consisting of red cell membrane (RCM), and the peptides TAT and PCM. Liposomes were prepared using a membrane dispersion-ultrasonic method; the prepared 1% TAT and 3% PCM micelles were mixed with liposomes and under overnight stirring to form polypeptid-modified liposomes. RCM was isolated from mice blood, and the mechanical force facilitated RCM adhesion to the lipid bilayer. The characteristics of liposomes such as the morphology, particle size, zeta-potential, and RCM-conjugation to lipsomes were evaluated. Uptake efficiency and cellular toxicity of liposomes were evaluated *in vitro* on myocardial cells (MCs). As regard the experiments *in vivo*, liposomes were intravenously injected into mice, and the blood and organs were collectedat different times to analyze the pharmacokinetics profile of liposomes. The cellular uptake and intracellular distribution of liposomes of different composition into MCs demonstrated that RCM-modified liposomes had the best delivery capability. The pharmacokinetics study further demonstrated that RCM-modified liposomes had prolonged mean residence time (MRT) and more accumulation in the heart. This study indicated that RCM can be used to modify liposomes in combination with polypeptides, because such modification increases the myocardial targeting of liposomes. Therefore, this system constructed in this study might be a potentially effective myocardial drug delivery system.

## Introduction

Among cardiovascular disease, ischemic cardiacdisease (ICD) is acoronary artery disease characterized by chronic or acute reduction of myocardial blood perfusion (Laflamme & Murry, 2011), and it is one of the most common causes of death worldwide. ICD is an important factor leading to hypoxic death and ventricular remodeling in cardiomyocytes, and the existing treatments cannot effectively prevent it. The conventional medical therapy is based on the coronary-artery bypass graft surgery, drug eluting stents and anti-thrombotic therapy (Spoladore et al., 2012; Won et al., 2014). Although significant efforts have been devoted to reduce the damage of these treatments, these damages cannot be ignored. Since the injury caused by ICD is manifested as remodeling of the myocardial tissue exacerbating the myocardial cell death, the treatments mentioned above cannot prevent the remodeling of myocardial tissue. Therefore, new treatments with better adaptability that can prevent myocardial remodeling should be developed. Compared with invasive treatments, a drug therapy exerts less damage to the body, although it is difficult to obtain the correct concentration of the drug in the myocardial tissue to obtain an effective result.

A number of advantages including good histocompatibility and cell affinity have been explored to prove the superiority of liposomes (Abu Lila et al., 2010; Rahman et al., 2017). Liposomes have a structure similar to the one of cells membranes, thus, they exert less damage to normal tissues and they have no inhibitory effects. Liposomes can also be digested by lysosomes to induce a natural drug release. However, despite the good biocompatibility of liposomes, some urgent unresolved problems with liposomes are still present, such as uncontrolled drugs release, drug leakage before reaching the intended cellular target, and body immune reaction. The polypeptide-modified liposomes are constructed to obtain liposomes with dual targeting, thereby achieving better efficacy and less side effects. PCM (WLSEAGPVVTVRALRGTGSW) is a peptide consisting of 20 amino acids selected by phage display technology, which can specifically bind to a matrix protein-tendon protein-X of cardiomyocytes (Barry et al., 1996; McGuire et al., 2004; Seiler, 2010). TAT is a cell penetrating peptide consisting of 11 amino acids (YGRKKRRQRRR), which can efficiently introduce various small and large molecules working as therapeutics (Yuan et al., 2015) such as a linked polypeptide and DNA into the cell without affecting the normal structure and function of the cells (Schwartz & Zhang, 2000; Torchilin, 2008). In our previous study, we demonstrated that PCM and TAT co-modified liposomes could improve their myocardial targeting ability (Wang et al., 2017).

The red cell membrane (RCM) has been identified as a promising alternative to drug delivery systems, as its high biocompatibility and low toxicity overcome the limitations of some synthetic polymers. In addition, it can be obtained from a wide range of different animals, with a relatively low cost, thus having broad application prospects. The large space within the red blood cells allows it to embed many drugs or proteins. RCM properties of RCM such as their structure and surface proteins were used as a starting point to design the next-generation delivery platforms (Tsai et al., 2010; Merkel et al., 2011). Using RCM to modify the nano-drug loading system can avoid the recognition by the immune system, with the significant advantage of prolonging the circulation of the drug in the body (Hu et al., 2012; Antonelli et al., 2016). RCM-modified nano-drug loading system can be used in combination with other treatment regimens, and have long circulating effects in targeted drug delivery systems (Muzykantov, 2013; Wu et al., 2016), photothermal therapy (Piao et al., 2014; Jiang et al., 2017), and tumor therapy (Gao et al., 2017; Rao et al., 2017). Consequently, our hypothesis was that RCM-modified liposomes might be a promising long-circulating drug delivery vehicle.

Therefore, in current study, a novel drug carrier for myocardium delivery was designed, composed of RCM attached to the polypeptide-modified liposomes. The liposomes consisted of soybean phospholipids (SPCs), cholesterol (CHO), DSPE-PEG_2000-MAL_. PCM and TAT were covalently coupled to the distal end of DSPE-PEG_2000-MAL_. The RCM was added to the polypeptides-modified liposomes. Coumarin-6 was encapsulated as a fluorescent probe for in vitro and in vivo studies. The characteristics of these modified liposomes were evaluated, such as their morphology, MCs uptake and intracellular distribution, and cytotoxicity in vitro. The targeting and pharmacokinetics of the drug delivery system was further verified in vivo by collecting the blood and hearts for high performance liquid chromatography (HPLC) analysis.

## Materials and methods

### Materials

SPCs were purchased from the American Jiaji Company (Minnesota, MN, USA). CHO and DSPE-PEG_2000-Mal_ were purchased from Shanghai Advanced Vehicle Technology L.T.D.Co (Shanghai, China). PCM and TAT peptide with terminal cysteine (WLSEAGPVVTVRALRGTGSW-Cys and AYGRKKRRQRRR-Cys) were synthesized by GL Biochem Ltd (Shanghai, China). Coumarin-6 and coumarin-7 were purchased from J&K Scientific LTD (Beijing, China). Penicillin–streptomycin was purchased from Gibco (California, USA).

### Animals

Male Kunming mice (20 ± 2 g) were purchased from the Experiment Animal Center of Chongqing Medical University, China. All animal experiments were performed at the Animal Experimental Center of Chongqing Medical University. The protocols were approved by the Animal Care and Use Committee of Chongqing Medical University. The animals were fed with a standard diet and access to water and food ad libitum for one week and kept in a laboratory environment at a temperature of 25 °C ± 2 before the start of the experiment.

### Preparation of liposomes

Coumarin-6 loaded conventional liposomes (L), coumarin-6 loaded TAT-modified liposomes (L-TAT), coumarin-6 loaded PCM-modified liposomes (L-PCM) andcoumarin-6 loaded TAT-PCM-modified liposomes (L-TAT-PCM) were prepared according to a previous study (Spoladore et al., 2012). In brief, liposomes were prepared by the thin-film hydration method, and the prepared 1% TAT and 3% PCM micelles were mixed with liposomes and under overnight stirring. The liposomes were dialyzed in massive PBS to remove the unbound peptides and coumarin-6.

The red cells were separated from the fresh blood of the mice by centrifugation (6000×*g*, 3 min). The precipitate was washed with PBS and treated with hypotonic PBS (0.025 mol/L), and then subjected to membrane rupture to remove the intracellular content. RCM were then collected by centrifugation (7000×*g*, 5 min). Liposomes (L, L-TAT, L-PCM, and L-TAT-PCM) and RCM were mixed at different volume ratio. After performing probe ultrasound in the water bath (150 W, 5 S, 5 S, 5 min), the mechanical force facilitated the adhesion of the RCM to the lipid bilayer, resulting in a vesicle-particle fusion. Then the fusion was dialyzed in massive PBS to remove the unbound RCM.

### Characterization of liposomes

To characterize the RCM-coated liposomes, the particles size, polydispersity index (PDI), and surface charge were measured using Zetasizer (Nano ZS, Malvern instrument, UK). THE morphology of liposomes was determined using Transmission Electron Microscopy (H-600IV, Hitachi, Japan). To determine the encapsulation efficiency (EE) of coumarin-6 loaded L, L-RCM, L-TAT, L-TAT-RCM, L-PCM, L-PCM-RCM, L-TAT-PCM, and L-TAT-PCM-RCM, 1 ml liposome suspension was purified by dialysis with PBS. After demulsification with methanol, the fluorescence intensity was observed by fluorospectrophotometry (Ex = 465 nm, Em = 502 nm). The EE was calculated by the ratio of mean intensity after and before dialysis.

Following the structural studies, the protein content of the liposomes examined to verify the conjugation of RCM. The RCM-coated liposomes were dialyzed with PBS for 24 h to remove unbound membranes and subsequently treated with RIPA to solubilize the membrane proteins. Samples of RCM were prepared in parallel as a comparison. Proteins were separated by sodium dodecyl sulfate-polyacrylamide gel electrophoresis (SDS-PAGE) and then stained with coomassie blue for 1 h. Then, the proteins were decolorized in a solution of (ethyl alcohol: acetic acid: H_2_O = 5:10:85, v:v:v) for 5–10 times. Images of each protein were captured by using a molecular imager (California, USA).

### Confocal laser microscopy

MCs were seeded in 24-well culture plates at a density of 4 × 10^4 ^cells/well, cultured in DMEM containing 10% FBS, and incubated at 37 °C, under 5% CO_2_ for 24 h to allow cell adherence. Then the culture medium was discarded and replaced with 100 μl serum-free DMEM containing different coumarin-6 loaded liposomes (L, L-RCM, L-TAT, L-TAT-RCM, L-PCM, L-PCM-RCM, and L-TAT-PCM-RCM), with a final coumarin-6 concentration of 0.25 μg/mL. After incubation for 1 h, the culture medium was removed, and cells were washed 3 times with sterile PBS. The cells were fixed using 4% paraformaldehyde for 15 min and then counter stained by DAPI for 10 min. Images of each treatment were captured using a Zeiss Elyra laser scanning confocal microscope (Tokyo, Japan).

### Flow cytometry

MCs in a logarithmic growth phase were seeded into 6-well plates at a density of 30 × 10^4^ cells/well, cultured in DMEM containing 10% FBS for 24 h and incubated at 37 °C, under 5% CO_2_. Then, the culture medium was removed, and replaced with 500 μl serum-free DMEM containing different coumarin-6 loaded liposomes (L, L-RCM, L-TAT, L-TAT-RCM, L-PCM, L-P-RCM, L-TAT-PCM-RCM), with a final coumarin-6 concentration of 1 μg/ml. The cells were incubated at 37 °C for 1 h, and the samples were then prepared for flow cytometry analysis. The cells were rinsed with PBS for three times, subjected to trypsinization, collected and centrifuged at 1000×*g* for 5 min. The supernatant was removed, and the pellet was resuspended in 200 µL PBS. Data for 10,000 events using an acquisition gate were collected and analyzed by a Beckman Coulter CytoFlex flow cytometer (Becton Dickinson, San Jose, CA). The results were analyzed using the FlowJo software (Becton, Dickinson and Company (BD), Warwick, RI, USA).

### In vitro cytotoxicity assay

In vitro cytotoxicity of different was detected by Cell Counting Kit-8 (CCK-8). MCs were seeded into a 96-well plate at a density of 4 × 10^4^ cells/well, cultured in DMEM containing 10% FBS, and incubated at 37 °C, under 5% CO_2_ for 24 h. Then the culture medium was discarded and replaced with 100 µl serum-free DMEM containing different coumarin-6 loaded liposomes (L, L-RCM, L-TAT, L-TAT-RCM, L-PCM, L-PCM-RCM, and L-TAT-PCM-RCM). Cells were incubated at 37 °C, 5% CO_2_ for 1 h. The viability of cells cultured in serum-free culture medium was set to 100%. The culture medium was removed and 100 μl CCK-8 diluted in PBS (v:v = 10:90) was added to each well. After 4 h incubation, the absorbance was read at a wavelength of 450 nm on a microplate reader (Thermo Scientific Varioskan Flash, Waltham, MA).

### In vivo experiments

A randomized design was applied to divide the mice into three groups with 40 animals in each group. The mice in the three groups were administrated with L, L-RCM (v:v = 20:1) and L-RCM (v:v = 10:1), respectively. Two hundred μl of different liposomes were injected into the mice tail vein in each group at a coumarin-6 dose of 1 mg/kg. Blood samples from each group were collected from the eyelids at specific time intervals of 15, 30, 60 min, 2, 4, 8, 12, 24, 48, and 72 h after tail vein injection. Blood was collected at each time point and placed in a centrifuge tube previously treated with sodium heparin, and then centrifuged at 5000×*g* for 5 min to separate the plasma from the blood. The plasma was collected and stored at −80 °C prior to further analysis.

In a separated experiment, another randomized design was applied to divide the mice into two groups with 60 animals in each group. The mice in the two groups were treated with TAT-PCM-L and TAT-PCM-L-RCM (v:v = 10:1), respectively. Two hundred μl of different liposomes were injected into the mice tail vein in each group at a coumarin-6 dose of 1 mg/kg. Blood was collected at each time point and centrifuged, and the plasma was collected and stored at −80 °C before further analysis. Mice hearts were harvested at 24 h, 48 h, and 72 h. Each organ was weighed, and 1 ml PBS was added, and the tissue was homogenized. The tissue homogenate was centrifuged at 6000×*g* for 5 min, and 200 μl supernatant was collected and stored at −80 °C before further analysis.

### HPLC assay

Biosample treatment: 10 ul coumarin-7 (1 μg/ml) were added to the plasma or the supernatant of the tissue homogenate. After intensive mixing, 1 ml *n*-hexane was added, followed by vortexing, and ultrasonicated to extract coumarin-6 and coumarin-7. The samples were centrifuged at 6000×*g* for 5 min, and the supernatant was collected and dried overnight in a drying oven under a constant temperature to remove the solvent. Hundred μl methanol were added to each sample before HPLC assay.

The HPLC assay was performed on a Waters Alliance E2695 HPLC System with a fluorescence detector. HPLC conditions were as follows: The 20 μl injection volume was run on a reverse-phase C18 (200 × 4.6 mm, 5 mm) column, and the mobile phase consisted of methyl alcohol and water (90:10, v/v) at a flow rate of 1.0 mL/min. The column temperature was maintained at 30 °C and the excitation and emission wavelength were 465 nm and 502 nm, respectively. This analytical method was verified to meet our methodological requirements.

### Statistical analysis

The concentration–time curve was used to determine the pharmacokinetic parameters. The main pharmacokinetic parameters were the mean residence time (MRT) and the area under the plasma concentration–time curve (AUC_0–∞_). The parameters were calculated using DAS 2.0 software and are expressed as mean ± standard deviation (SD).

All experiments were performed at least in triplicate and the results are expressed as mean ± SD. Comparisons were performed using one-way ANOVA, then Turkey’s post-hoc test. A *p*-value < 0.05 was considered statistically significant.

## Results

### Preparation and characterization of the liposomes

**Figure 1.**
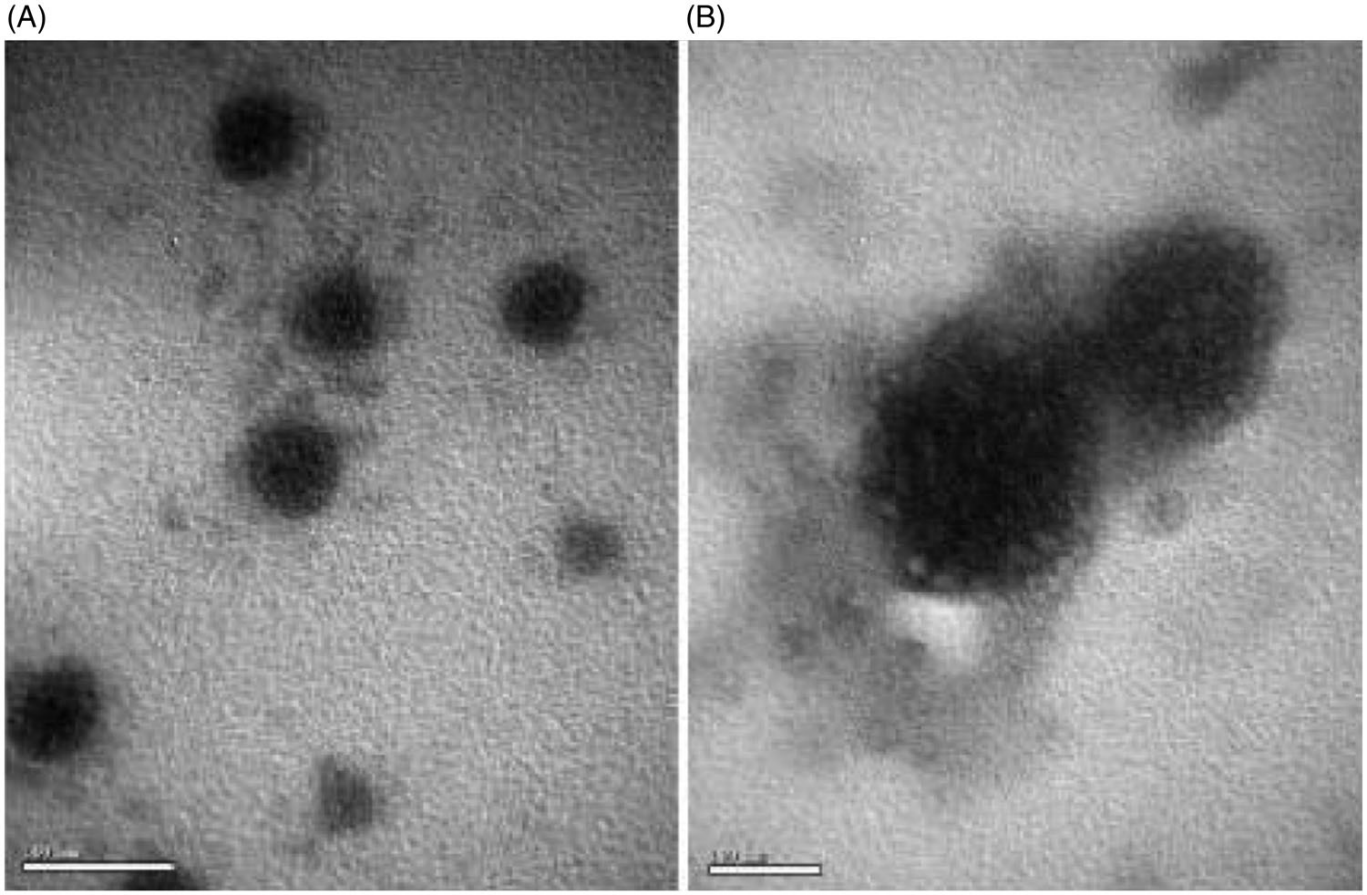
TEM images of coumarin-6 loaded TAT-PCM-modified liposomes (L-TAT-PCM) and RCM-modified TAT-PCM-modified liposomes (L-TAT-PCM-RCM). (A) L-TAT-PCM and (B) L-TAT-PCM-RCM.

The particlel size, PDI, Zeta potential and the EE of coumarin-6 different liposomes are shown in Table 1. The mean particle diameter of non-modified conventional liposome (L) was 108.7 ± 0.56 nm and RCM-modified liposomes (L-RCM) was 260 ± 0.60 nm. The zeta potential measurements showed that all liposomes bear an overall positive charge ranging from 15 to 17 mV. The liposomes incubated with the RCM exhibited a larger partical size. To characterize the non-modified liposomes and RCM-modified liposomes, the particlel were visualized using TEM. The TEM images (Figure 1) showed that PCM and TAT co-modified liposomes (L-TAT-PCM) and RCM co-modified liposomes (L-TAT-PCM-RCM) were spherical and regularly shaped. The electron microscopic images showed the attachment of RCM to the surface of the liposomes.

**Table 1.** Particle size, PDI, zeta-potentialand encapsu-lation efficiency of various liposomes.

Samples	Size(nm)	PDI	Zeta(mV)	EE/%
L	108.7 ± 0.56	0.141 ± 0.024	−15.0 ± 0.3	90.23 ± 2.03
L-RCM	260 ± 0.60	0.254 ± 0.027	−16.1 ± 0.3	89.67 ± 1.98
L-TAT	109 ± 0.47	0.174 ± 0.020	−15.3 ± 0.4	90.32 ± 2.54
L-TAT-RCM	278 ± 0.65	0.247 ± 0.013	−16.5 ± 0.3	90.53 ± 3.02
L-PCM	117 ± 0.50	0.168 ± 0.031	−15.1 ± 0.1	89.83 ± 2.56
L-PCM-RCM	282 ± 0.34	0.316 ± 0.015	−16.3 ± 0.3	88.45 ± 3.04
L-TAT-PCM	120 ± 0.68	0.198 ± 0.025	−15.2 ± 0.2	87.57 ± 2.39
L-TAT-PCM-RCM	290 ± 0.73	0.294 ± 0.035	−16.6 ± 0.3	87.57 ± 2.78

Results are expressed as mean ± SD (*n* = 3). L: coumarin-6 loaded coventional liposomes; L-RCM: RCM-coated liposomes; L-TAT: coumarin-6 loaded TAT-modified liposomes; L-TAT-RCM: RCM-coated TAT-modified liposomes; L-PCM: coumarin-6 loaded PCM-modified liposomes; L-PCM-RCM: RCM-coated PCM-modified liposomes; L-TAT-PCM: coumarin-6 loaded TAT-PCM-modified liposomes; L-TAT-PCM-RCM: RCM-TAT-PCM-modified liposomes.

**Figure 2.**
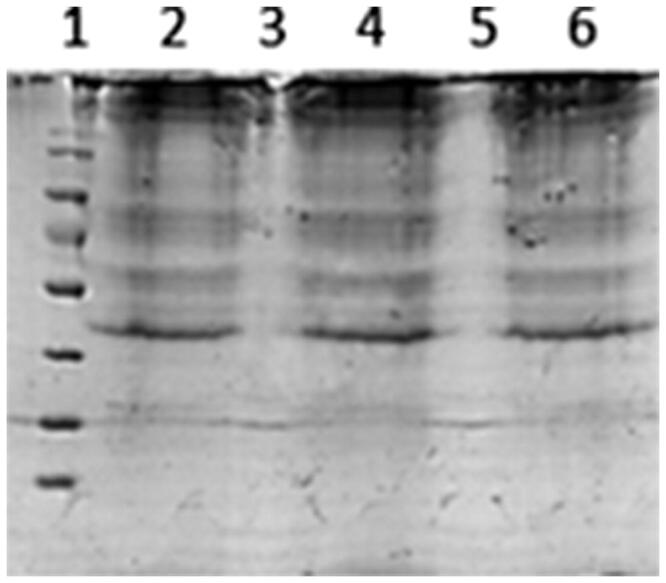
SDS-PAGE of liposomes with or without RCM. Line 1: RCM; Line 2: liposomes (L); Line 3: RCM-coated liposomes (L-RCM); Line 4: coumarin-6 loaded TAT-PCM-modified liposomes (L-TAT-PCM); Line 5: RCM-coated TAT-PCM-modifiedliposomes (L-TAT-PCM-RCM).

Protein separation by SDS-PAGE demonstrated that membrane proteins were present on the RCM modified liposomes, and were mostly retained in the liposomes preparation, as the protein bands of RCM modified liposomes were consistent with RCM. Liposomes without RCM have no protein bands (Figure 2).

### Qualitative and quantitative cellular uptake in vitro

**Figure 3.**
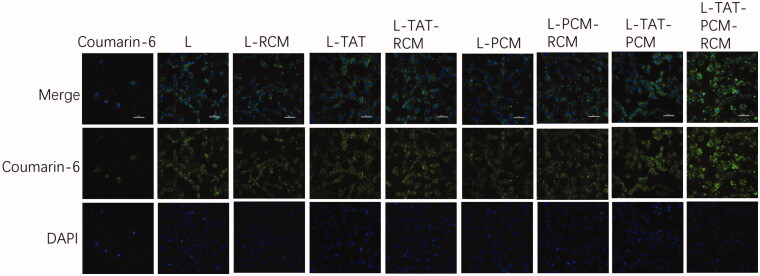
Cellular uptake of different liposomes by MCs. The green fluorescence is referred to the coumarin-6 after entering the cell, and the blue color indicates the nucleus stained by DAPI. L: coumarin-6 loaded conventional liposomes; L-RCM: RCM-coated liposomes; L-TAT: coumarin-6 loaded TAT-modified liposomes; L-TAT-RCM: RCM-coated TAT-modified liposomes; L-PCM: coumarin-6 loaded PCM-modified liposomes; L-PCM-RCM: RCM-coated PCM-modified liposomes; L-TAT-PCM: coumarin-6 loaded TAT-PCM-modified liposomes; L-TAT-PCM-RCM: RCM-TAT-PCM-modified liposomes.

**Figure 4.**
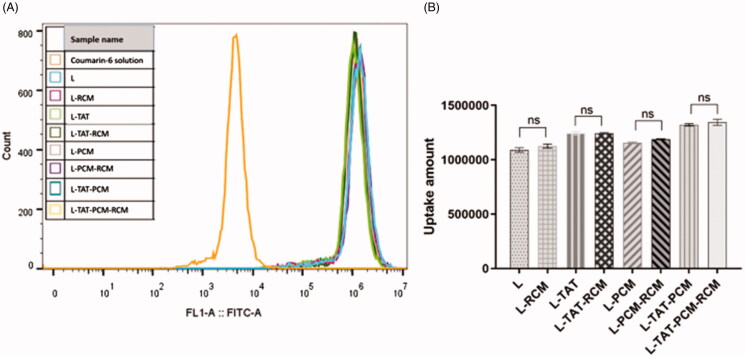
Fluorescence intensity of different liposomes by flow cytometry. The results are expressed as means ± SD (*n* = 3). L: coumarin-6 loaded conventional liposomes; L-RCM: RCM-coated liposomes; L-TAT: coumarin-6 loaded TAT-modified liposomes; L-TAT-RCM: RCM-coated TAT-modified liposomes; L-PCM: coumarin-6 loaded PCM-modified liposomes; L-PCM-RCM: RCM-coated PCM-modified liposomes; L-TAT-PCM: coumarin-6 loaded TAT-PCM-modified liposomes; L-TAT-PCM-RCM: RCM-TAT-PCM-modified liposomes.

To confirm the cellular uptake and the intracellular release of liposomes in MCs, the naturally fluorescent coumarin-6 was observed under a confocal laser scanning microscopy. DAPI was used to stain the nucleus. When the coumarin-6 solution was used, a sparse amount of green fluorescence was observed in the cytoplasm only. However, the fluorescence was clearly observed in the cytoplasm when coumarin-6 loaded liposomes were used (Figure 3). The fluorescence of coumarin-6 was not different when RCM-modified liposomes or liposomes without RCM were used. The cellular uptake of coumarin-6 was quantified by flow cytometry. The fluorescence intensity of MCs after incubation with coumarin-6 loaded liposome at a coumarin-6 concentration of 0.25 μg/mL showed that the mean fluorescence intensity of coumarin-6 in liposome-treated cells was no difference among different groups (Figure 4). The mean fluorescence intensity of liposomes was 240–250 times stronger compared with coumarin-6 in PBS.

### Cytotoxicity in vitro

**Figure 5.**
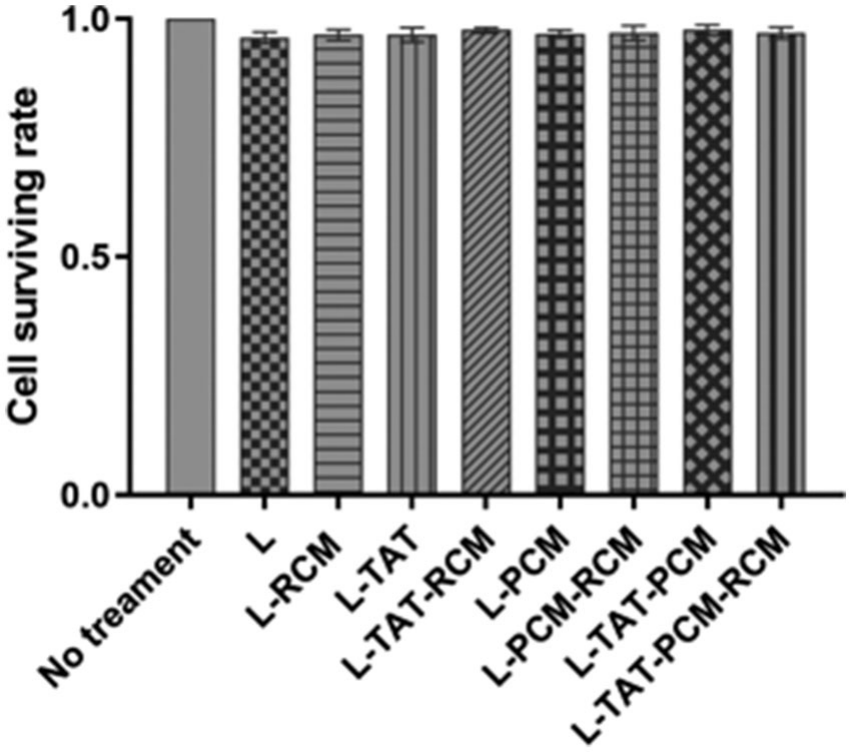
Cell viability in MCs after the treatment with different modified liposome. The results are expressed as means ± SD (*n* = 5). L: coumarin-6 loaded coventional liposomes; L-RCM: RCM-coated liposomes; L-TAT: coumarin-6 loaded TAT-modified liposomes; L-TAT-RCM: RCM-coated TAT-modified liposomes; L-PCM: coumarin-6 loaded PCM-modified liposomes; L-PCM-RCM: RCM-coated PCM-modified liposomes; L-TAT-PCM: coumarin-6 loaded TAT-PCM-modified liposomes; L-TAT-PCM-RCM: RCM-TAT-PCM-modified liposomes.

As shown in Figure 5, the results revealed that all liposomes were nontoxic to MCs at all concentrations, with a viability rate higher than 90% at 0.25 mg/mL.

### Pharmacokinetics study

**Figure 6.**
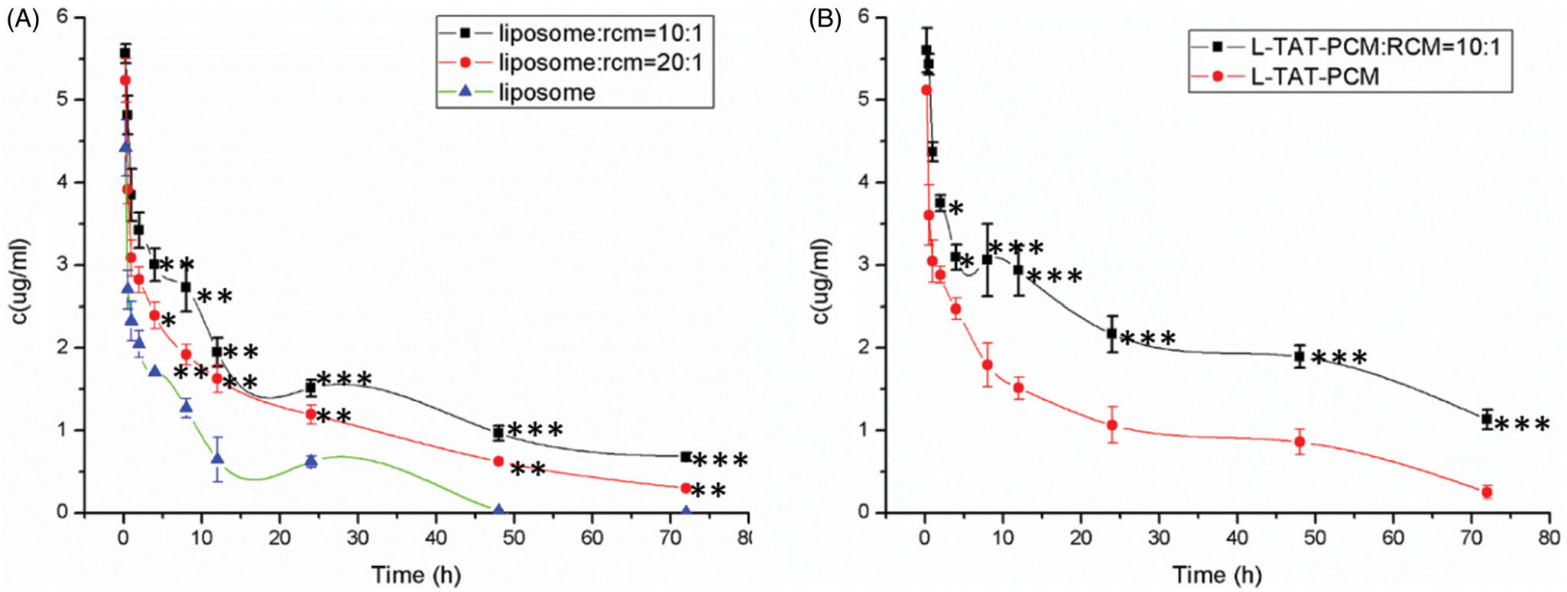
Plasma concentration of coumarin-6 after intravenous administration of different liposomes. Results are expressed as mean ± SD (*n* = 6). (A) Plasma concentration of coumarin-6 loaded L, RCM-L (1:10) and RCM-L (1:20). L: conventional liposomes; RCM-L (1:10): RCM modified conventional liposomes with a RCM/liposome ratio of 1:10 (v:v); RCM-L (1:20): RCM modified conventional liposomes with a RCM/liposome ratio of 1:20 (v:v). *p < 0.05; **p < 0.01; ***p < 0.001 compared with L. (B) Plasma concentration of coumarin-6 loaded L-TAT-PCM and RCM-L-TAT-PCM. L-TAT-PCM: coumarin-6 loaded TAT-PCM-modified liposomes; RCM-L-TAT-PCM (1:10): coumarin-6 loaded TAT-PCM-modified liposomes with a RCM/liposome ratio of 1:10 (v:v); *p < 0.05; **p < 0.01; ***p < 0.001 compared with L-TAT-PCM.

**Figure 7.**
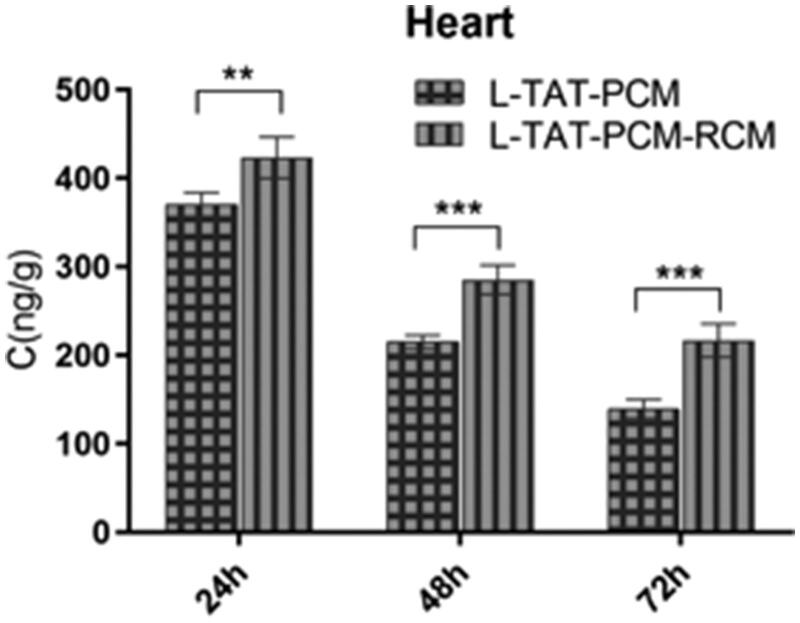
Concentration of coumarin-6 in the heart after administration of L-TAT-PCM and L-TAT-PCM-RCM at 24 h, 48 h, and 72 h. The results were expressed as the weight ratio of coumarin-6 and heart (ng/g) and presented as mean ± SD (*n* = 6).**p < 0.01; ***p < 0.001 compared with L-TAT-PCM.

The concentration–time curves are shown in Figure 6, and the main pharmacokinetic parameters are listed in Table 2. The results showed that the addition of RCM to liposomes could significantly prolong their blood circulation, with the MRT increased by 79.2% (L:RCM = 10:1, *p* < 0.01) and 27.3% (L:RCM = 20:1, *p* < 0.05) and the AUC in blood increased by 77.6% (L:RCM = 10:1, *p* < 0.001) and 137.3% (L:RCM = 20:1, *p* < 0.01), compared with L. Furthermore, the biodistribution of the liposomes in the heart was evaluated. The RCM modified liposomes showed a significant accumulation in heart tissue, with an AUC in the heart tissue increased by 100.1% (L:RCM = 10:1, *p* < 0.01, Table 3); and the coumarin-6 concentration in the heart tissue increased by 16.3% (*p* < 0.01) at 24 h, 30.2% (*p* < 0.001) at 48 h and 51.0% (*p* < 0.001) at 72 h after injection (Figure 7).

**Table 2.** MRT and AUC of coumarin-6 after intravenous administration of coumarin-6 loaded L, RCM-L (1:10) and RCM-L (1:20).

	L	RCM: L = 1：20	RCM: L = 1：10
MRT (h)	10.811 ± 1.466	13.761 ± 1.201*	19.378 ± 2.103***
AUC (mg/L*h)	33.377 ± 7.587	59.289 ± 9.678**	79.197 ± 9.623***

Results are expressed as mean ± SD (*n* = 6). L: conventional lipsomes; RCM-L (1:10): RCM modified conventional liposomes with a RCM/liposome ratio of 1:10 (v:v); RCM-L (1:20): RCM modified conventional liposomes with a RCM/liposome ratio of 1:20 (v:v). *p < 0.05; **p < 0.01; ***p < 0.001 compared with L.

**Table 3.** MRT and AUC of coumarin-6 loaded L-TAT-PCM and RCM-L-TAT-PCM.

	L-TAT-PCM	RCM: TAT-PCM-L = 1：10
MRT (h)	12.067 ± 1.391	22.846 ± 2.462***
B-AUC (mg/L*h)	107.142 ± 11.121	158.071 ± 14.251*
H-AUC (mg/L*h)	192.749 ± 16.755	402.001 ± 21.631***

L-TAT-PCM: coumarin-6 loaded TAT-PCM-modified liposomes; RCM-L-TAT-PCM (1:10): coumarin-6 loaded TAT-PCM-modified liposomes with a RCM/liposome ratio of 1:10 (v:v); *p < 0.05; ***p < 0.001 compared with L-TAT-PCM.

## Discussion

ICD is a cardiovascular disease with high mortality. The current treatments mainly focus on restoring the blood circulation in the ischemic area. Although the ischemic state of myocardium is significantly improved, the recovery of blood circulation also causes serious reperfusion injury, further damaging the MCs in the ischemic area and causing serious myocardial remodeling. Therefore, it is necessary to develop a new myocardial targeting drug delivery system which can effectively accumulate in the ischemic myocardial area and in which the drug carrier can be uptaken by the MCs.

Several myocardium-affinity ligands were used to combine them with liposomes form myocardial targeting. In our previous study, a dual-ligand liposome delivery system composed of PCM and TAT co-modified liposomes (L-TAT-PCM) was developed. This liposome was able to effectively deliver therapeutic drugs to the myocardium for the treatment of myocardial ischemia. To further increase the myocardial targeting of the system, the system was modified with the RCM. RCM is natural, nontoxic, and biocompatible and it can be dissociated and dissolved in the organism’s natural conditions. It was previously demonstrated that the RCM has a promising use in modifying nanoparticles (Hu et al., 2014; Chen et al., 2016; Fang et al., 2017; Sun et al., 2019). By taking advantage of the ‘maker-of-self’ protein CD47 on the RBC surface, RCM modified nanoparticles emerged as a strategy to help nanoparticles to avoid the recognition by the immune system, thus prolonging the circulation time of nanoparticles (Oldenborg, 2000). RCM coatings attracted significant attention due to the natural features derived from their source cells. A top-down strategy was used to develop a RCM-coating nanoplatform, in which the natural functions of existing cells can be directly transferred to make the ultimate nanomedicine (Narain et al., 2017). In addition, several studies considered blood cell membrane-coated NPs that resulted beneficial in cancer chemotherapy (Bose et al., 2018; Jiang et al., 2019). Some researcher explored natural melanin nanoparticles extracted from living cuttlefish as effective photothermal agents and developed RCM-camouflaged melanin (Melanin@RBC) nanoparticles as a platform for in vivo antitumor PTT (Jiang et al., 2017). Qin Jiang successfully fused RCM together with MCF-7 cell membrane and fabricated erythrocyte-cancer hybrid membrane-camouflaged melanin nanoplatform Melanin@RBC-M for in vivo photothermal therapy (Jiang et al., 2019). Aryal et al. reported RCM-coated, DOX-loaded poly (lactic acid) (PLA) NPs. In this study, the authors compared two strategies, physical encapsulation and chemical conjugation, for loading DOX into the PLA NPs (Aryal et al., 2013; Muzykantov, 2013). Hu CM et al. reported a top-down biomimetic approach in particle functionalization by coating biodegradable polymeric nanoparticles with natural erythrocyte membranes, including both membrane lipids and associated membrane proteins for long-circulating cargo delivery (Hu et al., 2011). Nanoparticles coated with RCM have been found to retain the binding adhesion molecules on their surfaces, allowing a high targeting to homologous cancer cells. However, these studies focused on tumor cells, while both RCM and peptides were used in this work to target the heart. In addition, nanoparticles coated with red cell membrane have been found to retain the binding adhesion molecules on their surfaces, allowing a high targeting to homologous cancer cells. However, these studies focused on tumor cells, while both RCM and peptides were used in this work to target the heart. In addition, in the present study, RCM was, for the first time, successfully conjugated to liposomes.

The structure, size surface zeta potential, and protein contents of the RCM-coated liposomes indicated that the RCM might conjugated to the liposomes, suggesting that a novel myocardial targeted drug delivery system was formed. The particles of RCM-derived vesicles were examined for their protein contents, and the results showed that the functionality of the membrane-associated proteins was retained (Hu et al., 2011). The proteins in RCM were successfully associated to the liposomes. It should also be noted that the structure of the RCM was retained throughout the entire preparation process to minimize the loss of and damages to the membrane proteins. Thus, these results predicted that the modified liposomes could exhibit a long circulation time with the help of these proteins.

The results of pharmacokinetics study demonstrated that comparing with the RCM-modified liposomes possessed significant prolonged circulation time, with higher coumarin-6 concentrations in plasma and heart tissue at the same time points compared with L-PCM-TAT without RCM modification. The results of were consistent with the results of other RCM-related studies performed on cancer therapy (Piao et al., 2014; Gao et al., 2017). In addition, the AUC and MRT of the RCM-modified liposomes were significantly increased compared with the unmodified liposomes. These results further validated that the RCM modification could effectively help liposomes to prolong their circulation time and accumulating in the heart tissue.

To verify whether RCM modification influences the uptake of liposomes by MCs, in vitro uptake assay was qualitatively and quantitatively performed. All coumarin-6 loaded liposomes exhibited evident green fluorescence of coumarin-6 in the cytoplasm. TAT and PCM co-modified liposomes (L-TAT-PCM) showed the strongest fluorescence intensity compared with TAT or PCM-modified and non-modified liposomes. This result was consistent with our previous study, and indicated that the co-modifition with peptides could improve the cell uptake of liposomes by MCs. The same uptake features of RCM modified liposomes were observed, comparing with the corresponding liposomes without RCM. The quantification assay showed the same trend. These results indicated that the presence of RCM had no influence in the uptake of liposomes by MCs.

As a new class of vector extracted from living organisms, the obtained RCM-liposomes would be biocompatible with less toxicity (Fang et al., 2018; Liu et al., 2019). Indeed, our results showed that the survival rate of cells reached as high as 94.69% when treated with RCM-liposomes and showed with no significant difference when liposomes without RCM were used. This result indicated that the presence of RCM had no influence on the cytotoxicity of liposomes on MCs. Both blank liposomes and RCM were biocompatible and low toxic.

In conclusion, a novel RCM-modified dual-ligand liposomes delivery system was developed in present study, providing better cardiomyocyte targeting and long-circulating ability, thus able to deliver therapeutic drugs to the myocardium more efficiently for the treatment of myocardial ischemia.

## References

[CIT0001] Abu Lila AS, Ishida T, Kiwada H, et al. (2010). Targeting anticancer drugs to tumor vasculature using cationic liposomes. Pharm Res 27:1171–83. 20333455 10.1007/s11095-010-0110-1

[CIT0002] Antonelli A, Sfara C, Weber O, et al. (2016). Characterization of ferucarbotran-loaded RBCs as long circulating magnetic contrast agents. Nanomedicine (Lond) 11:2781–95. 27739933 10.2217/nnm-2016-0216

[CIT0003] Aryal S, Hu CMJ, Fang RH, et al. (2013). Erythrocyte membrane-cloaked polymeric nanoparticles for controlled drug loading and release. Nanomedicine (Lond) 8:1271–80. 23409747 10.2217/nnm.12.153

[CIT0004] Barry MA, Dower WJ, Johnston SA, et al. (1996). Toward cell-targeting gene therapy vectors: selection of cell-binding peptides from random peptide-presenting phage libraries. Nat Med 2:299–305. 8612228 10.1038/nm0396-299

[CIT0005] Bose RJ, Paulmurugan R, Moon J, et al. (2018). Cell membrane-coated nanocarriers: the emerging targeted delivery system for cancer theranostics. Drug Discov Today 23:891–9. 29426004 10.1016/j.drudis.2018.02.001

[CIT0006] Chen Z, Zhao P, Luo Z, et al. (2016). Cancer cell membrane-biomimetic nanoparticles for homologous-targeting dual-modal imaging and photothermal therapy. ACS Nano 10:10049–57. 27934074 10.1021/acsnano.6b04695

[CIT0007] Fang RH, Jiang Y, Fang JC, et al. (2017). Cell membrane-derived nanomaterials for biomedical applications. Biomaterials 128:69–83. 28292726 10.1016/j.biomaterials.2017.02.041 PMC5417338

[CIT0008] Fang RH, Kroll AV, Gao W, et al. (2018). Cell membrane coating nanotechnology. Adv Mater 30:e1706759. 29582476 10.1002/adma.201706759 PMC5984176

[CIT0009] Gao M, et al. (2017). Erythrocyte-membrane-enveloped perfluorocarbon as nanoscale artificial red blood cells to relieve tumor hypoxia and enhance cancer radiotherapy. Adv Mater 29:1701429. 10.1002/adma.201701429 28722140

[CIT0010] Hu CMJ, Fang RH, Luk BT, et al. (2014). Polymeric nanotherapeutics: clinical development and advances in stealth functionalization strategies. Nanoscale 6:65–75. 24280870 10.1039/c3nr05444f

[CIT0011] Hu CMJ, Fang RH, Zhang L, et al. (2012). Erythrocyte-inspired delivery systems. Adv Healthc Mater 1:537–47. 23184788 10.1002/adhm.201200138

[CIT0012] Hu CMJ, Zhang L, Aryal S, et al. (2011). Erythrocyte membrane-camouflaged polymeric nanoparticles as a biomimetic delivery platform. Proc Natl Acad Sci U S A 108:10980–5. 21690347 10.1073/pnas.1106634108 PMC3131364

[CIT0013] Jiang Q, Liu Y, Guo R, et al. (2019). Erythrocyte-cancer hybrid membrane-camouflaged melanin nanoparticles for enhancing photothermal therapy efficacy in tumors. Biomaterials 192:292–308. 30465973 10.1016/j.biomaterials.2018.11.021

[CIT0014] Jiang Q, Luo Z, Men Y, et al. (2017). Red blood cell membrane-camouflaged melanin nanoparticles for enhanced photothermal therapy. Biomaterials 143:29–45. 28756194 10.1016/j.biomaterials.2017.07.027

[CIT0015] Laflamme MA, Murry CE. (2011). Heart regeneration. Nature 473:326–35. 21593865 10.1038/nature10147 PMC4091722

[CIT0016] Liu B, Wang W, Fan J, et al. (2019). RBC membrane camouflaged prussian blue nanoparticles for gamabutolin loading and combined chemo/photothermal therapy of breast cancer. Biomaterials 217:119301. 31279101 10.1016/j.biomaterials.2019.119301

[CIT0017] McGuire MJ, Samli KN, Johnston SA, et al. (2004). In vitro selection of a peptide with high selectivity for cardiomyocytes in vivo. J Mol Biol 342:171–82. 15313615 10.1016/j.jmb.2004.06.029

[CIT0018] Merkel TJ, Jones SW, Herlihy KP, et al. (2011). Using mechanobiological mimicry of red blood cells to extend circulation times of hydrogel microparticles. Proc Natl Acad Sci U S A 108:586–91. 21220299 10.1073/pnas.1010013108 PMC3021010

[CIT0019] Muzykantov VR. (2013). Drug delivery carriers on the fringes: natural red blood cells versus synthetic multilayered capsules. Expert Opin Drug Deliv 10:1–4. 23176316 10.1517/17425247.2013.750292

[CIT0020] Narain A, Asawa S, Chhabria V, et al. (2017). Cell membrane coated nanoparticles: next-generation therapeutics. Nanomedicine (Lond *)* 12:2677–92. 28965474 10.2217/nnm-2017-0225

[CIT0021] Oldenborg PA. (2000). Role of CD47 as a marker of self on red blood cells. Science 288:2051–4. 10856220 10.1126/science.288.5473.2051

[CIT0022] Piao JG, Wang L, Gao F, et al. (2014). Erythrocyte membrane is an alternative coating to polyethylene glycol for prolonging the circulation lifetime of gold nanocages for photothermal therapy. ACS Nano 8:10414–25. 25286086 10.1021/nn503779d

[CIT0023] Rahman M, Beg S, Verma A, et al. (2017). Therapeutic applications of liposomal based drug delivery and drug targeting for immune linked inflammatory maladies: a contemporary view point. Curr Drug Targets 18:1558–71. 28413980 10.2174/1389450118666170414113926

[CIT0024] Rao L, Cai B, Bu LL, et al. (2017). Microfluidic electroporation-facilitated synthesis of erythrocyte membrane-coated magnetic nanoparticles for enhanced imaging-guided cancer therapy. ACS Nano 11:3496–505. 28272874 10.1021/acsnano.7b00133

[CIT0025] Schwartz JJ, Zhang S. (2000). Peptide-mediated cellular delivery. Curr Opin Mol Ther 2:162–7. 11249637

[CIT0026] Seiler C. (2010). The human coronary collateral circulation. Eur J Clin Invest 40:465–76. 20534067 10.1111/j.1365-2362.2010.02282.x

[CIT0027] Spoladore R, Maron MS, D'Amato R, et al. (2012). Pharmacological treatment options for hypertrophic cardiomyopathy: high time for evidence. Eur Heart J 33:1724–33. 22719025 10.1093/eurheartj/ehs150

[CIT0028] Sun D, Chen J, Wang Y, et al. (2019). Advances in refunctionalization of erythrocyte-based nanomedicine for enhancing cancer-targeted drug delivery. Theranostics 9:6885–900. 31660075 10.7150/thno.36510 PMC6815958

[CIT0029] Torchilin VP. (2008). Tat peptide-mediated intracellular delivery of pharmaceutical nanocarriers. Adv Drug Deliv Rev 60:548–58. 18053612 10.1016/j.addr.2007.10.008

[CIT0030] Tsai RK, Rodriguez PL, Discher DE, et al. (2010). Self inhibition of phagocytosis: the affinity of ‘marker of self’ CD47 for SIRPalpha dictates potency of inhibition but only at low expression levels. Blood Cells Mol Dis 45:67–74. 20299253 10.1016/j.bcmd.2010.02.016 PMC2878922

[CIT0031] Wang X, Huang H, Zhang L, et al. (2017). PCM and TAT co-modified liposome with improved myocardium delivery: in vitro and in vivo evaluations. Drug Deliv 24:339–45. 28165817 10.1080/10717544.2016.1253121 PMC8241121

[CIT0032] Won YW, Bull DA, Kim SW, et al. (2014). Functional polymers of gene delivery for treatment of myocardial infarct. J Control Release 195:110–9. 25076177 10.1016/j.jconrel.2014.07.041 PMC4252832

[CIT0033] Wu YW, Goubran H, Seghatchian J, et al. (2016). Smart blood cell and microvesicle-based Trojan horse drug delivery: Merging expertise in blood transfusion and biomedical engineering in the field of nanomedicine. Transfus Apher Sci 54:309–18. 27179926 10.1016/j.transci.2016.04.013

[CIT0034] Yuan M, Qiu Y, Zhang L, et al. (2015). Targeted delivery of transferrin and TAT co-modified liposomes encapsulating both paclitaxel and doxorubicin for melanoma. Drug Delivery 23:1171–83. 26036724 10.3109/10717544.2015.1040527

